# Immunogenicity, efficacy, and safety of human papillomavirus vaccine: Data from China

**DOI:** 10.3389/fimmu.2023.1112750

**Published:** 2023-03-13

**Authors:** Mingzhu Li, Chao Zhao, Yun Zhao, Jingran Li, Lihui Wei

**Affiliations:** Department of Obstetrics and Gynecology, Peking University People’s Hospital, Beijing, China

**Keywords:** immunogenicity, efficacy, safety, human papillomavirus vaccine, China

## Abstract

The incidence rate and mortality rate of cervical cancer have steadily increased in young women in China. Therefore, it is critical to improve HPV vaccination rates, particularly for the younger population. There are currently five types of prophylactic vaccines in China: bivalent HPV vaccine (AS04-HPV-16/18), quadrivalent HPV vaccine, 9-valent HPV vaccine, homemade Escherichia coli-produced HPV bivalent vaccine, and Pichia pastoris produced HPV bivalent vaccine. All these five HPV vaccines have completed relevant clinical trials in China, and have been proven to be generally well-tolerated and immunogenic, efficacious against persistent HPV-related infections and genital precancerous lesions (data for 9-valent HPV vaccine is absent), and have demonstrated acceptable safety profiles, as previously shown in global studies. Given that the HPV vaccination rate in China is still very low, additional HPV vaccine coverage is needed to reduce the incidence and mortality rates of cervical cancer.

## 1 Introduction

Cervical cancer is the leading cause of malignant tumors in women. In 2020, there were appropriately 604,000 cases of cervical cancer worldwide, resulting in 342,000 deaths, and making for an 88.1% incidence and 91.4% mortality rate in low- and middle-income countries (1). These rates are expected to rise in China. The annual incidence and mortality rate of cervical cancer in China significantly increased from 2000 to 2016 based on the 2020 International Agency for Research on Cancer report, with 109,741 new cases and 59,060 deaths, accounting for 18.2% of global new cases and 17.3% of global deaths, respectively (2).

In China, it is estimated that the national overall high-risk prevalence of human papillomavirus (hrHPV) infection is 15.54% (95% CI: 13.83%-17.24%) (3). The five most common types of HPV in the general population are HPV-16 (3.52%, 95% CI: 3.18%-3.86%), HPV-52 (2.20%, 95% CI: 1.93%-2.46%), HPV-58 (2.10%, 95% CI: 1.88%-2.32%), HPV-18 (1.20%, 95% CI: 1.05%-1.35%), and HPV-33 (1.02%, 95% CI: 0.89%-1.14%) (3). Of these, HPV-16 and HPV-18 are extremely common and together account for approximately 84.5% of squamous cell carcinoma cases (4). A “two-peak” phenomenon for the highest hrHPV infection rate was observed in the 15-19-year-old group and the 50-60-year-old group (5). The prevalence of cervical cancer among patients ≤ 35 years old increased from 2.8% to 15.7% in the past 30 years in Hubei, China (6), indicating that it is critical to improve HPV vaccination coverage, particularly for the younger population.

Three types of HPV vaccines are currently used worldwide: a bivalent (bv) HPV vaccine (AS04-HPV-16/18), a quadrivalent (qv) HPV vaccine, and a 9-valent (9v) HPV vaccine. All three vaccines are licensed by the U.S. Food and Drug Administration (FDA), are recognized by the World Health Organization (WHO), and have been approved for use in China’s female population. Two independently developed bv-HPV vaccines have been approved by the National Medical Products Administration (NMPA) in China. In total, there are currently five types of HPV vaccines that can be used in Chinese females (**Table 1**), all of which have completed relevant clinical trials in China.

**Table 1:** Prophylactic HPV vaccine approved for use in China.

Vaccine	bv-HPV vaccine (AS04-HPV-16/18)	qv-HPV vaccine	9v-HPV vaccine	bv-HPV vaccine (E.c)	bv-HPV vaccine (P.p)
Name	Cervarix	Gardasil	Gardasil-9	Cecolin	Walrinvax
Manufacturer	GSK, UK	MSD, USA	MSD, USA	Xiamen Innovax, China	Walvax, China
Expression system	*Baculovirus*	*Saccharomyces cerevisiae*	*Saccharomyces cerevisiae*	*Escherichia coli*	*Pichia pastoris*
Approved time (Global/China)	2007/2016	2006/2017	2014/2018	-/2019	-/2022
HPV types prevented	HPV16/18	HPV 6/11/16/18	HPV 6/11/16/18/ 31/33/45/52/58	HPV 16/18	HPV 16/18
Age for vaccination (China)	9~45y	9~45 y	9~45 y	9~45 y	9~30 y
Vaccination schedule	0 M,1 M, 6 M *	0 M,2 M, 6 M	0 M,2 M, 6 M	0 M,1 M, 6 M *	0 M,1 M, 6 M *
Site of inoculation	Intramuscular injection, upper arm deltoid preferred				
HPV-related disease control (approved by China)	HPV16/18 related CIN1, CIN2/3, AIS and cervical cancer	HPV16/18 related CIN1, CIN2/3, AIS and cervical cancer	HPV16/18/31/33/45/52/58 related CIN1, CIN2/3, AIS and cervical cancer	HPV16/18 persistent infection, HPV16/18 related CIN1, CIN2/3, AIS, and cervical cancer	HPV16/18 related CIN1, CIN2/3, AIS and cervical cancer

* 9–14 y: two doses, 0 M, 6 M;

CIN, cervical intraepithelial neoplasia; AIS, adenocarcinoma *in situ*.

## 2 Immunogenicity

### 2.1 Bivalent HPV (AS04-HPV-16/18) vaccine

A randomized, controlled, double‐blind study (NCT00779766) of bv-HPV (AS04-HPV-16/18) vaccine was evaluated according‐to‐protocol efficacy (ATP‐E) cohort in healthy Chinese females aged 18‐25 years(vaccine, n= 2888; control, n= 2892). At Month 72, >95% women(n= 664) in the ATP cohort remained seropositive for anti‐HPV16 and HPV18 antibodies, the geometric mean titers (GMTs) were 678.1 EU/mL (95% CI: 552.9, 831.5) and 343.7 EU/mL (95% CI: 291.9, 404.8), respectively (7). By comparing with this clinical trial, other Phase IIIB, randomized, controlled trials(NCT00996125 and NCT01277042) of the bv-HPV (AS04-HPV-16/18) vaccine was evaluated in healthy Chinese females aged 9-45 years, which received vaccine (9-17 years, n = 374; 26-45 years, n = 606) and control (9-17 years, n = 376; 26-45 years, n = 606) at months 0, 1, and 6, the result found that immune responses at month 7 were non-inferior for 26-45-year-old women compared to 18-25-year-old women. The GMT was 2-3-fold higher in 9-17-year-old girls than in 18-25-year-old women. Furthermore, GMTs for both anti-HPV16 and anti-HPV18 antibodies in the youngest age groups (9–11 and 12–14 y) were higher comparing with the higher-aged groups (8).

### 2.2 Escherichia coli-produced HPV bivalent vaccine

A randomized, immunogenicity noninferiority study (NCT02562508) of HPV bivalent vaccine in Escherichia coli (E coli), termed bv-HPV vaccine (E.c), was conducted in China, which include girls aged 9-14 years (2 doses, n=301; 3 doses, n=304), 15-17 years (3 doses, n=149) and women aged 18-26 years (3 doses, n=225). The statistical results demonstrated that the antibody levels of HPV16 and 18 at month 7 decreased as age increased, the GMT levels of antibodies for both HPV-16 (95% CI:1.76;1.56, 1.99) and HPV-18 (95% CI:1.93; 1.69, 2.21) in girls receiving 3 doses were noninferior to those in women receiving 3 doses. In the 2-dose 9-14 year old group, 100% and 98.6% of girls seroconverted for IgG to HPV-16 and HPV-18 in the sixth month, respectively, the antibody level to HPV-16 was noninferior to that in girls who received 3 doses, with GMT (95% CI:0.70; 0.63, 0.79) for IgG, however, the GMT noninferiority for HPV-18 was not proven (9). PPS-I (representing ‘a per-protocol sets for immunogenicity’) of a phase-III trial (NCT01735006) and an immune-bridging trial (NCT02562508) in China found that bv-HPV vaccine (E.c) could induce a specific antibody response in women aged 9-45-years-old, and that antibody levels are negatively correlated with age. Additionally, there was a declining trend for HPV16 and HPV18 in >35-year-old women (10). A slight deviation in the vaccination time of the 2^nd^ and 3^rd^ doses had little effect on the immune response (11).

### 2.3 Pichia pastoris produced HPV bivalent vaccine

A Phase III-IIIB, randomized, controlled trials (311-HPV-1003 and 311-HPV-1004) of HPV bivalent vaccine in Pichia pastoris ((bv-HPV(P.p)) in 9-30 year aged females showed that the GMT of HPV16 and 18 serum in girls aged 9-17 years was higher than that in women aged 18-30 years, and the neutralizing antibodies of HPV16 and 18 were all above 99.77% in both age groups. The neutralizing antibodies in girls receiving 2 doses were noninferior to that in women aged 18-26 who received 3 doses (the lower limit of 95%CI for GMT ratio was greater than 0.67, and the lower limit of 95%CI for positive difference was greater than -5%) (see instruction book).

### 2.4 Quadrivalent HPV vaccine

A randomized, double-blind, placebo-controlled trial of the quadrivalent HPV vaccine (qv-HPV vaccine) among 9–15 year old Chinese males (n = 100) and 9–45 year old Chinese females (n = 500) from Wuzhou, Guangxi, China, demonstrated that one month after the third dose, seroconversion rates for HPV6, 11, 16 and 18 were 96.7% (95% CI: 93.7, 98.5), 99.3% (95% CI: 97.4, 99.9), 99.3% (95% CI: 97.4, 99.9), and 99.0% (95% CI: 97.0, 99.8) in vaccine group, respectively. Males and females aged 9–15 had a 1.4–2.8-fold higher immune response to vaccination than females aged 16–26 years old (12).

### 2.5 9-valent HPV vaccine

Regarding 9v-HPV vaccine immunogenicity, a phase-III open-label study (NCT03903562) among 1990 females (9-19 years, n=690; 20-26 years, n=650; 27-45 years, n=650) demonstrated that 99% of participants seroconverted to the corresponding vaccine for their HPV type after one month when they received 3 doses(100% seroconversion of all participants, with the exception of HPV18 in 20–26-year old group (99.8% seroconversion)). The anti-HPV6/11/16/18/31/33/45/52/58 seroconversion percentages in 9-19-year-old and 27-45-year-old Chinese females were not inferior than those of 20-26 years old females (13). Based on these results, the NMPA approved extending the age of the 9v-HPV vaccine for 9-45-year-old women in August 2022.

## 3 Efficacy

### 3.1 Bivalent HPV (AS04-HPV-16/18) vaccine

A phase II/III, randomized, controlled trial (NCT00779766.) was conducted among 18-25-year-old Chinese women of the bv-HPV (AS04-HPV-16/18) vaccine. Efficacy was assessed in the according‐to‐protocol efficacy (ATP‐E) cohort (vaccine, n= 2888; control N = 2892), total vaccinated cohort for efficacy (TVC‐E) (vaccine, n= 2987; control n = 2985) and TVC‐naïve (vaccine, n= 1660; control, n=1587). Vaccine efficacy (VE) against HPV‐16/18 related CIN2 or worse was 87.3% (95% CI: 5.5, 99.7) in the ATP‐E, 88.7% (95% CI: 18.5, 99.7) in the TVC‐E, and 100% (95% CI: 17.9, 100) in the TVC‐naïve cohort(all 14 high-risk types of HPV DNA were negative, and HPV 16 and 18 were seronegative negative and cytologically negative at baseline) (7).

### 3.2 Escherichia coli-produced HPV bivalent vaccine

An interim analysis of a randomized clinical trial (NCT01735006) of bv-HPV vaccine (E.c)among 7372 eligible women aged 18–45 in China showed that the VE against high-grade genital lesions(HSILs) and persistent infections (PIs) associated with HPV-16/18 was 100.0% (95% CI:55.6% to 100.0%; vaccine:0/3306 vs control: 10/3296) and 97.8% (95% CI: 87.1% to 99.9%,1/3240 vs 45/3246) in the per-protocol cohorts, respectively (14). A phase III, double-blind, randomized, controlled trial(NCT01735006) from five study sites in China among 1455 women (vaccine: n=3689; control: n=3683) aged 18-45 years showed that after a long-term follow-up (66 months), bv-HPV vaccine (E.c) still high VE against HSILs (100%;95% CI: 67.2-100.0;vaccine:0/3310 vs control: 13/3302) and PIs caused by HPV16 and HPV18 (97.3%;95% CI:89.9-99.7;2/3262 vs 73/3271) (15).

### 3.3 Pichia pastoris produced HPV bivalent vaccine

The Phase III, randomized, controlled trials (311-HPV-1003) of bv-HPV(P.p) vaccine in 18-30 year aged females showed that the efficacy for HPV16/18 related cervical intraepithelial neoplasia 2(CIN2+) was 78.6% (95% CI: 23.3, 96.1; vaccine:3/5190 vs control: 14/5167) in PPS-I population after follow-up at 48 months (see instruction book).

### 3.4 Quadrivalent HPV vaccine

During a phase-III randomized, double-blind, placebo-controlled, multi-center clinical trial (NCT00834106) of the qv-HPV vaccine in China, efficacy for HPV16/18-related CIN2+ and HPV6/11/16/18-related CIN 1+ was 100% (95% CI: 32.3, 100; 0 vs 7 cases) and 100% (95% CI: 70.9, 100; 0 vs 14 cases) in the per-protocol efficacy (PPE) population of 20-45-year-old women after follow-up at 78 months. The efficacy against 6-month PIs was 91.6% (95% CI: 66.0, 99.0) at Month 30, and that against 12-month PIs was 97.5% (95% CI: 85.1, 99.9) at Month 78 (16). A recently published 8-year follow-up on the efficacy of the qv-HPV vaccine among 368 participants demonstrated that 3-dose continued protection of the qv-HPV vaccine against HPV-related diseases lasted a median of 94 months, with the longest time being 125 months in 20-45-year-old Chinese women(vaccine:0**/**8 vs control: 2**/**19) (17).

### 3.5 Women with HPV infections

A post-hoc analysis of a phase II/III study (NCT00779766) evaluated the efficacy of bv-HPV (AS04-HPV-16/18) VE against hrHPV infections in 871 Chinese women aged 18-25-years over a 72-month follow-up period. Its results demonstrated that the VE against 6-month and 12-month HPV-16/18 PIs in women DNA-negative to HPV-16/18 but DNA-positive to any other HR-HPV type at baseline was 100.0% (95% CI: 79.8-100.0) and 100.0% (95%CI: 47.2-100.0), respectively. The VE against HPV-31/33/45 incident infections, in women DNA-positive to HPV-16/18 and DNA-negative to other HPV types at baseline was 71.0% (95%CI: 27.3-89.8). This indicates that women with existing HR-HPV infections at the time of vaccination could still benefit from the AS04-HPV-16/18) vaccine (18).

### 3.6 Women with excisional treatment

A post-hoc analysis focused on intent-to-treat in 168 participants(vaccine, n = 87; placebo, n = 81) who received excisional treatments at baseline and during a follow-up period in a phase II/III, double-blind, randomized trial (NCT00779766) conducted in Jiangsu Province, China, found that the VE of vaccination on acquiring 14 hrHPV infection after treatment was 27% (95% CI: 4.9, 44.0%).VE against new infections after treatment for 14 hrHPV infection was estimated as 32.0% (95%CI:1.8, 52.8%), and was 41.2% (95%CI:162.7, 86.8%) for HPV16/18 infection. Although no significant evidence demonstrated that the bv-HPV (AS04-HPV-16/18) vaccine could lead to faster viral clearance(vaccine:88.9% vs control: 81.6%) or have any effect on the rates of persistent infection among women who received excision treatments, the post-treatment women could still experience benefits due to the “primary prophylactic” effect (19).

## 4 Safety

A phase I trial (NCT00549900) assessing the bv-HPV (AS04-HPV-16/18) vaccine demonstrated that pain at the injection site was the most commonly reported symptom. Two participants reported medically significant adverse incidents, but the investigator found that they were not related to vaccination (20). The per-protocol cohort of bv-HPV (E.c) vaccine study (NCT01735006) showed mild side effects, pain at the injection site (34.0%) and fever (>37.0°C, 35.1%) were the most common reactions that occurred in the vaccine group. No vaccine-related medical adverse incidents were observed (14). The end-of-study analysis also demonstrated a similar rate of serious adverse events between vaccine groups (7.2%, 267 of 3691) and the control (7.9%, 290 of 3681), and was not considered to be vaccination-related (15).

A safety assessment of the qv-HPV vaccine was performed in a clinical trial (NCT03903562) with Chinese women during a 90-month follow-up period and found that injection-site adverse events (AEs) 15 days after vaccination were common among qv-HPV vaccine participants (37.6% vs. 27.8%), and that systemic AEs had a similar frequency between the qv-HPV vaccine and placebo groups (46.8% vs. 45.1%). Pregnancy outcomes, fetal/infant SAEs, and new medical conditions in the qv-HPV vaccine and placebo groups were similar (21). The phase III open-label study(NCT03903562) compared the 9-valent HPV vaccine in 9-45-year-old Chinese females demonstrated that injection-site AEs were common such as injection-site pain (39.2%,45.2%, and 39.8%) and injection-site erythema (9.3%, 11.2%, and 7.4%) in the 9–19-year, 20–26-year, and 27–45-year age groups, respectively. No SAEs, or related discontinuations, or deaths were confirmed to be vaccine-related (13).

Although the above clinical trials in China all proved that all HPV vaccines can be tolerated, demonstrate immunogenicity and efficacy against HPV-related infections and genital precancerous lesions (data for 9-valent HPV vaccine is absent), and have acceptable safety profiles as previously shown in global studies, the rate of HPV vaccination in China still must be improved. According to 2021 survey data, only 3% of all Chinese females and 1.9% of females aged 9-14 received the HPV vaccine (22). The Chinese National Health Commission has proposed to gradually launch free HPV vaccinations nationwide to protect women and girls from cervical cancer, starting in pilot regions. At present, many regions in China are actively promoting free HPV vaccination by implementing the ‘Healthy China 2030 Program Outline’. For primary prevention, it is necessary to strengthen public health guidance and promote the administration of cervical cancer vaccines. Administering the HPV vaccine to adolescent women is a top priority since effectively preventing cervical cancer requires receiving the HPV vaccine before they become sexually active.

## Author contributions

LW designed the study, CZ conducted personnel organization and data collection, YZ and JL reviewed the literature, and ML drafted the manuscript. All authors contributed to the article and approved the submitted version.
